# Infant's DNA Methylation Age at Birth and Epigenetic Aging Accelerators

**DOI:** 10.1155/2016/4515928

**Published:** 2016-12-12

**Authors:** Ruheena Javed, Weidan Chen, Fangqin Lin, Huiying Liang

**Affiliations:** ^1^Institute of Pediatrics, Guangzhou Women and Children's Medical Center, Guangzhou Medical University, Jinsui Road 9, Tianhe District, Guangzhou, Guangdong 510623, China; ^2^Department of Cardiac Surgery, Guangzhou Women and Children's Medical Center, Guangzhou Medical University, Jinsui Road 9, Tianhe District, Guangzhou, Guangdong 510623, China

## Abstract

Knowing the biological age of the neonates enables us to evaluate and better understand the health and maturity comprehensively. However, because of dearth of biomarkers, it is difficult to quantify the neonatal biological age. Here we sought to quantify and assess the variability in biological age at birth and to better understand how the aging rates before birth are influenced by exposure in intrauterine period by employing a novel epigenetic biomarker of aging (epigenetic clock). We observed that the methylation age at birth was independent of the infant's sex but was significantly influenced by race. Partial correlation analysis showed a significant negative relationship between maternal socioeconomic status and infants' methylation age (*r*_*s*_ = −0.48, *P*_*s*_ = 0.005). A significant association with the risk of fast aging was observed for prenatal exposure to tobacco smoke with OR (95% CI) of 3.17 (1.05–9.56). Both estimated cell abundance measures and lymphocyte subpopulations in cord blood showed that tobacco exposed group exhibit an altered T cell compartment, specifically substantial loss of naive T cells. Present study provides the first evidence that common perinatal exposure (such as maternal smoking and lower socioeconomic status) may be important aging accelerators and substantial loss of naive T cells may play a role in the smoking-related fast aging phenomenon.

## 1. Introduction

In the broadest sense, we are aging from the moment of birth until the moment of death. But the rate and manner of aging vary markedly among individuals. It is a simple fact that, almost everywhere in the world, women live longer than men. You cannot use someone's chronological age to diagnose their health status; measuring symptoms of age-associated disease is not the same as measuring ageing. Rather than chronological age, your “biological” age is what is going to determine when you show clinical symptoms of disease. Such observations have led to the search for molecular markers of age which can be used to predict, monitor, and provide further insight into age-associated physiological decline and disorders [1]. One such marker is leukocyte telomere length, a molecular trait strongly correlated with biologic aging, which has been shown to have an accelerated rate of decay under environmental stress such as smoking [2, 3]. Furthermore, recently published study provides the first evidence to demonstrate a positive association between shortened fetal telomere length and smoking during pregnancy in a dose-response pattern [4].

All these in turn suggest two plausible hypotheses: (i) the aging processes begin before birth [5], and (ii) exposure to certain chemical substances in intrauterine life might increase/decrease the risk of at least some of pregnancy-related disorders through hastening/delaying the aging process [6]. But there were insufficient evidences to accept or reject a causal connection and that recommended a comprehensive research program, because assessing of the biological age of fetus and newborn infant poses significant methodological challenges. Biological age determines the time of clinical disease symptoms and which “age-associated” disease will depend on your genetic, epigenetic, and environmental risks factors (and of course those random factors).

Epigenetics comes from the Greek term “epi” meaning on or around the gene. In simple terms it is a mechanism that describes how genes can be switched on or off by chemical signals, a bit like a dimmer switch on a light, without altering the DNA sequence. These signals can alter the way genes produce proteins or signal other genes and, importantly, they can last months or years and are potentially reversible. These epigenetic switches are triggered by many factors such as our lifestyle, environment, and our age and, as the development of a growing fetus in the womb is totally dependent on these signals, it can alter the function of its cells. This can come in many forms and shows just how important these finely controlled mechanisms are for normal life. The one most easily studied is methylation; biological aging is reflected by highly reproducible DNA methylation changes at specific sites in the genome.

Excitingly, three recent studies used methylation measures from CpG sites across the genome to predict chronological age in humans [17–19]. Hannum et al. [17] created an age predictor based on 71 CpG sites in which DNA methylation was measured in whole blood. Weidner et al. [19] built a quantitative model of aging using DNA methylation values of just 3 CpG sites. Horvath et al. [18] developed an age predictor (“epigenetic clock”) using DNA methylation data from multiple studies (including the Hannum dataset) and multiple tissues. This clock is defined as a prediction method of chronological age based on the DNA methylation levels of 353 CpG sites. The predicted age resulting from the epigenetic clock runs alongside (*r* ≈ 0.96) but not always in parallel with chronological age. The difference between predicted age and chronological age (Δ_age_) can be put forth as an index of disproportionate “biological” aging and was hypothesized to be associated with risk for age-related diseases and mortality [20]. To data, however, no study has tried to determine whether neonatal biological ages can be quantified and compared and test whether DNA methylation-based Δ_age_ at birth is a significant predictor of prenatal adverse exposure.

Here, we adopted a novel epigenetic biomarker of aging (referred to as an “epigenetic clock”) to quantify and assess the variability in biological age among newborns and to better understand how the aging rates before birth are influenced by genetic and environmental exposure in intrauterine period.

## 2. Materials and Methods 

### 2.1. Public DNA Methylation Datasets

All genome-wide human methylation datasets from cord blood available in public data repositories at the time of analysis were used. Other DNA origins were also examined although, due to their poorer predictive accuracy (such as buccal cells [21], placentas [22], and special cell type from cord blood [23]), they were not included for the main analyses. Thus, all datasets used in present study consist of cord blood samples from newborns. Details on the individual dataset can be found in Table 1 (datasets 1–10). To give credit to the many researchers who generated the data, I briefly mention relevant citations. Datasets 1–5 consisting of cord bloods from 502 newborns were measured on the Illumina 27K array, and datasets 6–10 from 111 term born neonates were measured on Illumina 450K array.

### 2.2. Covariate Measurement

In dataset 1 [7], demographic data on the mothers (maternal ages) and newborns (gender and ethnicity) were abstracted from clinical records or reported by the mothers via mail questionnaires and phone calls during the months after delivery of the newborn. For dataset 2 [8], mothers' treatment status was used as covariates in the statistical models. Treatment status was dichotomized as placebo and micronutrient supplement [containing a balanced combination of 14 vitamins and minerals, specially formulated for use in pregnancy—Vitamin A (800 RE), Vitamin D (200 IU), Vitamin E (10 mg), Vitamin C (70 mg), Vitamin B1 (1.4 mg), Vitamin B2 (1.4 mg), Niacin (18 mg), Vitamin B6 (1.9 mg), Vitamin B12 (2.6 mg), Folic acid (400 *μ*g), Iron (30 mg), Zinc (15 mg), Copper (2 mg), Selenium (65 g), and Iodine (150 *μ*g)]. In dataset 3 provided by Turan and Sapienza [9] (GEO GSE36812), birth weight and gestational age information can be publicly available. In dataset 4 [10], prenatal smoke exposure status was assessed by two categories according to the cord blood cotinine levels: low cotinine and high cotinine. In dataset 5 [11], based on maternal self-report, the samples consist of 112 African-Americans, 91 European-Americans, and 13 other racial or mixed race groups. For dataset 8 reported by Hughes et al. [14] (GEO GSE54399), birth weight data were collected on newborns. In dataset 9 [15], tobacco exposure in utero was assessed by cotinine determination in the infants' umbilical cord blood: higher than 10 ng/mL was qualified as exposed to in utero tobacco and lower than 1 ng/mL was considered as nonexposed to in utero tobacco. For dataset 10, Sen et al. [16] randomly selected umbilical cord blood from 24 male and 24 female children from the 1st (<1.74 ng/dL) and 4th (>3.77 ng/dL) quartiles of Pb levels. Other variables were also determined: gender, categorical smoking status (yes/no), socioeconomic score (SES), gestational age, and birth weight. In datasets 6 [12], 7 [13], and 8, however, no other special covariates can be available besides gender of the newborns.

### 2.3. Estimated Blood Cell Percentages and Naïve T cell Abundance

In five Illumina 450k datasets (datasets 6–10), we also considered the proportions of CD8 T cells, CD4 T cells, natural killer cells, and B cells, and the abundance of defined different subtypes of T cells: naive T cells = CD45RA+CCR7+ and memory T cells = CD45RO+.

### 2.4. Novel Replication Samples

#### 2.4.1. Study Participants

Tobacco smoke exposed and nonexposed mothers and their newborns were recruited from those born in the Guangzhou Women and Children Medical Center between January 2012 and October 2015, using a convenient sampling technique of pregnant women attending the hospital for delivery. Only Han Chinese origin and born at term (gestational weeks 37 and 42) mothers were included. All subjects were born from uncomplicated pregnancies without any underlying medical conditions or medications and gave informed parental consent to participate. The study was approved by the review board of the Guangzhou Women and Children Medical Center and was carried out in accordance with the Declaration of Helsinki.

#### 2.4.2. Questionnaire Information

Mothers were asked to complete a standardized questionnaire including their smoking habit and passive exposure to tobacco smoke during pregnancy. If they were smokers, the average number of cigarettes smoked during pregnancy was recorded. Regarding exposure to secondhand smoke (SHS), mothers were asked if they were regularly exposed to SHS in the home, work, or vehicle and number of persons smoking in the home.

In utero tobacco smoke exposure of newborns was determined through maternal self-report and confirmed by cotinine levels in umbilical cord. Umbilical cord blood samples were obtained from the clamped umbilical cord immediately after delivery.

#### 2.4.3. Cotinine Concentrations

The cotinine level in serum from cord blood was analyzed by the method described by Xie et al. [24] using enzyme immunoassay technique (Immunalysis Corp., Catalogue # 233-0056; manufacturer reported detection limit, 1 ng/mL). The exposed to in utero tobacco group was defined by both a history of tobacco smoke exposure before pregnancy and by positive cotinine concentrations >3 ng/mL from cord blood [25]. Subjects who reported themselves to be nonsmokers with a cotinine level in umbilical cord lower than 1 ng/mL were considered as nonexposed to in utero tobacco. Additionally, subjects were selected for the nonexposed group based on sex distribution and mothers' age.

#### 2.4.4. Lymphocyte Characterization

Lymphocyte subsets in cord blood were measured on the BD FACSCalibur Flow Cytometer and analyzed using matching Cell Quest software according to the manufacturer's instructions. Lymphocyte subsets were categorized according to following definitions: T cells (CD3^+^), B cells (CD19^+^), NK cells (CD16^+^/CD56^+^), naïve (CD45RA^+^CCR7^+^CD27^+^) CD4^+^ or CD8^+^ T cells, and memory (CD45RO^+^) CD4^+^ or CD8^+^ T cells.

### 2.5. Statistical Analyses

The epigenetic clock is defined as a prediction method of age based on the DNA methylation levels of 353 CpG sites common to the Illumina 27K and 450K methylation arrays. Epigenetic age was calculated in all datasets using the online calculator (http://labs.genetics.ucla.edu/horvath/dnamage/) as reported previously [18]. A methylation-based age acceleration index (Δ_age_) was calculated for all newborns, defined as the methylation age in years minus chronological age at sample collection in years (Δ_age_ = epigenetic age – chronological age = epigenetic age – 0 = epigenetic age). Thus, the newborn who exhibits positive Δ_age_ was defined to be fast aging.

In five Illumina 450k datasets (datasets 6–10), the epigenetic clock software also allowed us to determine the ordinal abundance measures, proportions of CD8+ T cells, CD4+ T cells, natural killer cells (NK cells), and B cells, and different subtypes of T cells, naive T cells (CD45RA^+^CCR7^+^) and memory and effector T cells (CD8+CD28–CD45RA–). The statistical method for estimating these cell abundance measures was a penalized regression model (elastic net) to regress cell count measures (dependent variable) on DNA methylation levels. By applying this resulting penalized regression model to our data, we arrived at predicted cell abundance measures. Mathematical details and software tutorials for the epigenetic clock can be found in the supplemental files of [18].

Data are presented as means with SE for continuous variables and percentages for categorical variables. Significance of difference was determined by the nonpaired student's *t*-test and chi-square test. Spearman's rank test was used to investigate any possible correlation between variables. Unconditional logistic regression (stepwise) models were developed, and odds ratios (ORs) were used to evaluate risk factors associated with positive age acceleration of newborns. *P* values below 0.05 were considered significant. Statistical analysis was performed using Statistics 19.0 software (IBM SPSS Inc., Chicago, Ill).

## 3. Results

### 3.1. Altered Methylation Age among Newborns and Birth Prevalence of Fast Aging

**Figure 1:**
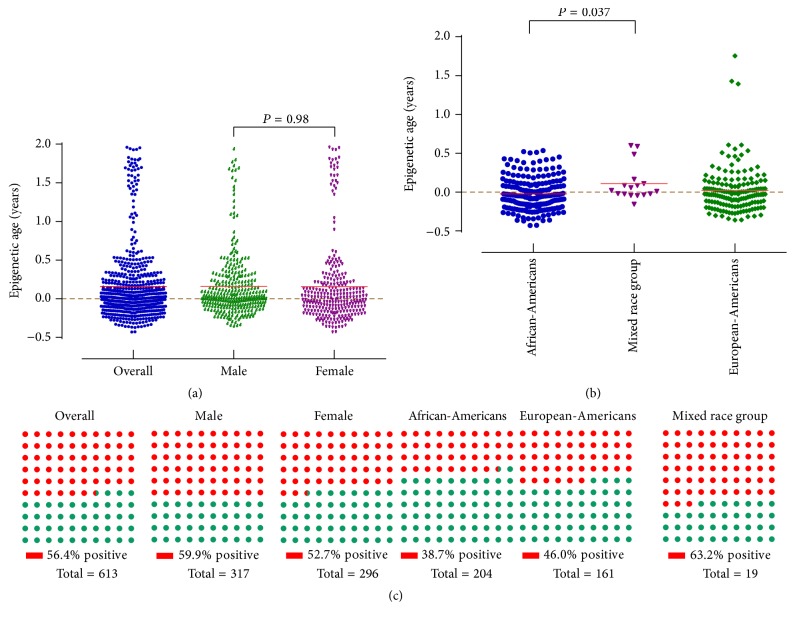
Epigenetic age and the prevalence of fast aging at birth of infants by gender and ethnicity.

Methylation age was calculated from a total of 613 cord blood samples profiled with the Illumina Infinium 450K (*n* = 111) and 27K (*n* = 502) arrays (Table 1). In the meta-analyzed results across the 10 datasets, the mean methylation ages at birth of the newborns were 0.16 ± 0.47 years: 0.16 ± 0.42 years and 0.16 ± 0.53 years for males and females, respectively (*P* = 0.98) (Figure 1). Using data from the datasets 1 and 5, African-American newborns had the lowest methylation age (−0.02 ± 0.21 years) compared with their European-American (0.02 ± 0.29 years) and mixed race (0.11 ± 0.23 years) counterparts (Figure 1).

Fast aging defined by a detectable methylation age of >0 years was present in 56.4% of babies as shown in Figure 1. Male infants as a group had higher prevalence of fast aging compared with female infants at birth (59.9% versus 52.7%). By race, the birth prevalence of fast ageing was highest in mixed race group (63.2%) followed by European-Americans (46.0%) and then African-Americans (38.7%).

### 3.2. Fetal and Maternal Factors Associated with Methylation Age and Fast Aging

**Figure 2:**
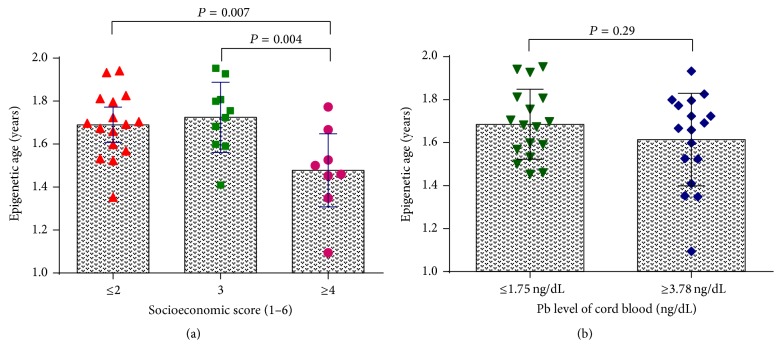
Comparison of the means of epigenetic age at birth in male and female newborns (a) and in groups with different levels of lead exposure (b).

Using data from dataset 10, we conducted partial correlations between SES and methylation age at birth, controlling for gender, birth weight, and lead exposure status. Lower SES was associated with higher methylation age, *r*_*s*_ = −0.48 and *P*_*s*_ = 0.005. The one-way ANOVA test revealed the same findings as in partial correlations. The average methylation ages were statistically significant lower in SES ≥ 4 group (1.48 ± 0.20 years) than that in both SES = 3 group (1.72 ± 0.16 years, *P* = 0.004) and SES ≤ 2 group (1.69 ± 0.15 years, *P* = 0.007) (Figure 2). Regarding lead exposure status, however, compared to the 1st quartile group of Pb, the 4th quartile group showed no significant change in methylation age at birth (Figure 2).

**Figure 3:**
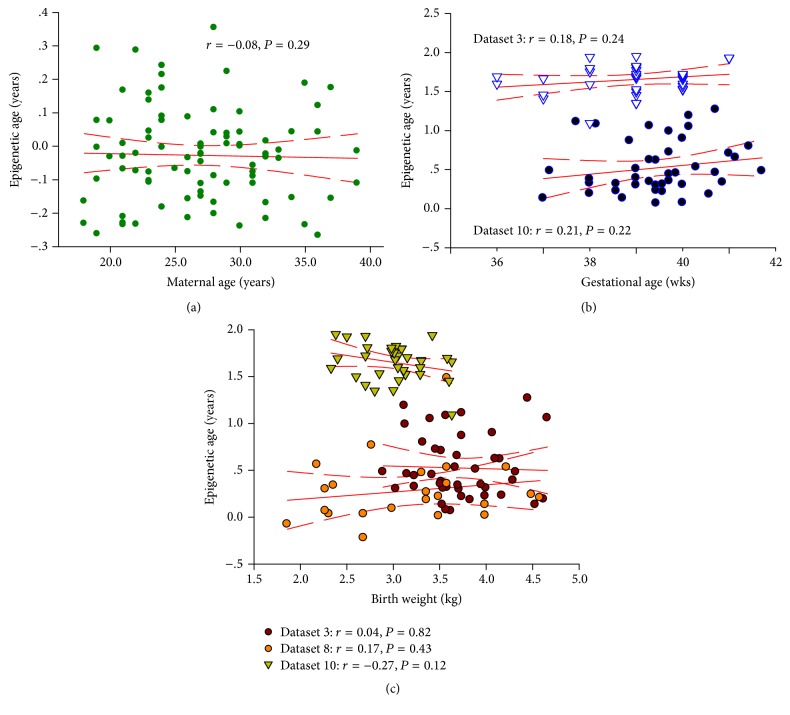
Analysis of the correlation of DNA methylation age to maternal age (a), gestational age (b), and birth weight (c) across the publicly available datasets.

The linear regression model showed no significant associations between maternal age (dataset 1: *r* = −0.08, *P* = 0.29), gestational age (dataset 3: *r* = 0.18, *P* = 0.24; dataset 10: *r* = 0.21, *P* = 0.22), and birth weight (dataset 3: *r* = 0.04, *P* = 0.82; dataset 8: *r* = 0.17, *P* = 0.43; dataset 10: *r* = −0.27, *P* = 0.12) with methylation age at birth (Figure 3).

Univariate logistic regression analyses were also conducted to explore the risk factors associated with fast aging (positive age acceleration). As shown in Table 2, the odds of positive age acceleration at birth for the mixed race group were more than twice those of African-Americans (OR = 2.71, 95% CI = 1.02–7.18). When African-Americans were compared with European-Americans, however, a similar trend was noted (OR = 1.35, 95% CI = 0.89–2.05). Using data from dataset 4, 9, and 10, infants of smoking mothers were more at increased risk for positive age acceleration (86.4%, OR = 3.17; 95% CI = 1.05–9.56) when compared to infants of nonsmoking mothers (66.7%). In dataset 2, although there were no significant differences between the groups in the percentage of infants with fast aging, there was a trend toward a lower incidence of fast aging in the group receiving pre- and periconceptual multiple micronutrient supplementation (52.4% versus 68.6%) (Table 2).

### 3.3. Effects of Tobacco Smoking during Pregnancy on Estimated Cell Abundance Measures in Umbilical Cord Blood

**Figure 4:**
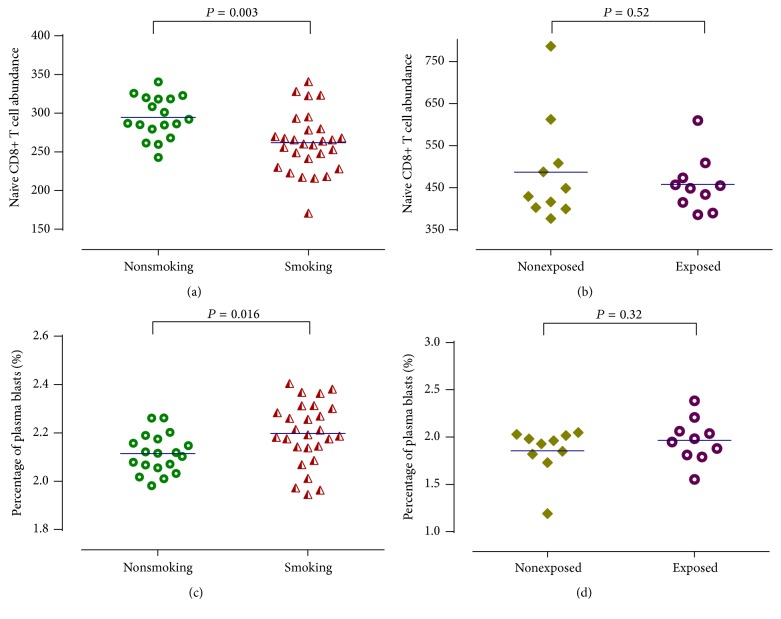
Relationship between prenatal tobacco exposure and the various cell abundance measures. Dataset 10: (a) and (c). Dataset 9: (b) and (d).

In dataset 9, infants exposed to tobacco in utero had higher proportion of plasma blasts (Figure 4) and lower naïve CD8+ T cell abundances (Figure 4) compared with nonexposed group at birth, although this difference is not statistically significant. Then, we confirmed these phenomenons in another independent dataset (dataset 10) as shown in Figure 4. The difference between newborns of nonsmoking and smoking mothers was statistically significant by *t*-test for means of estimated abundance measures of naïve CD8+ T cells (294.50 ± 6.31 versus 261.82 ± 7.26, *P* = 0.003) and proportion of plasma blasts (2.11 ± 0.02 versus 2.20 ± 0.02, *P* = 0.016) (Figure 4).

There were, however, no significant differences in other cell abundance measures between exposed and nonexposed groups including naïve CD4+ T cells, memory and effector T cells, proportion of CD8+ T cells, proportion of CD4+ T cells, proportion of NK cells, and proportion of B cells (Figure S1 in Supplementary Material available online at http://dx.doi.org/10.1155/2016/4515928).

### 3.4. Replication and Validation of Nicotine Exposure Related Lymphocyte Subpopulations Changes in Umbilical Cord Blood

**Figure 5:**
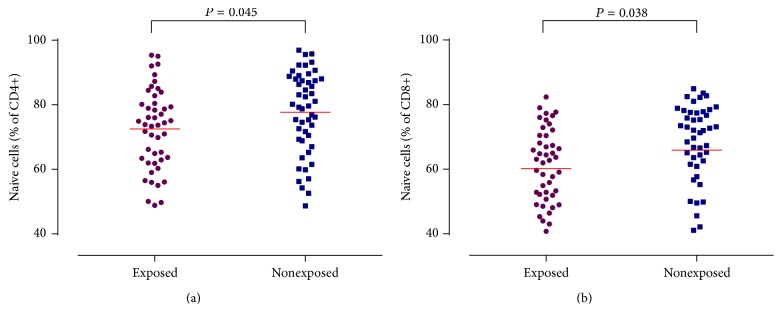
Proportions of naïve CD4+ and CD8+ T cells in newborns of smoking and nonsmoking mothers.

To replicate the nicotine exposure related lymphocyte subpopulations changes in umbilical cord blood, 47 nicotine exposed mother-newborn dyads and 49 nonexposed dyads with cord blood nicotine concentration confirmation were recruited. Analyses of the lymphocyte subpopulations of their cord blood samples revealed clear alterations in the CD4+ T cell compartment as well as in the CD8+ T cell compartment. We found significantly decreased proportion of naïve CD4+ (72.53 ± 1.80 versus 77.71 ± 1.82, *P* = 0.045) and naïve CD8+ T cell (60.22 ± 1.76 versus 65.90 ± 2.04, *P* = 0.038) counts in exposed group compared with nonexposed group (Figure 5). There were no significant differences between nonexposed group and exposed group regarding other lymphocyte subpopulations (Fig. S2).

## 4. Discussion

Knowing the biological age of the neonates enables us to evaluate the infant maturity and health status comprehensively. Quantifying estimation of neonatal age can help better understand the effects of epidemiological factors of pregnancy outcomes [26]. However, biological aging of fetus and infants is currently still a bigger challenge. Present study provides novel evidence that genome-wide DNA methylation-based analysis may provide a potential solution to this question as (i) the set of epigenetic modifications to DNA including DNA methylation and histone modifications has been related to chronological age over a long time scale and (ii) changes in methylation have been shown to play a key role in the development of age-related diseases [27].

But the factors that drive changes in the methylation age of adults and that constrain those changes are not well understood, let alone fetus and infants within the maternal intrauterine environment. However, we can reasonably attribute them to at least two underlying factors. First, it is possible that intrauterine environmental exposure will be associated with activation of epigenetic mechanisms leading to predictable and consistent changes in the epigenome. For instance, arsenic exposure before birth has been shown to decrease DNA methylation in boys [28] and in utero stress exposure to natural disaster has been associated with a broad, functional, and long-lasting DNA methylation signature in many kinds of tissues in offspring [29]. Alternatively, genetically determined epigenetic changes may also occur under or not under environmental pressure, inducing fundamentally unpredictable and personal-specific differences in the epigenome. For example, studies have reported some genetic variants (i.e., methylation quantitative-trait loci) regulating the methylation of age-associated markers [30]. Therefore, methylome states that quantitative measurements of infants at birth may identify factors related to slowed or accelerated rates of aging during intrauterine period.

As methylation age of infants at birth may be sensitive to a lot of factors, present study included several variants, such as gender, ethnicity, maternal age, gestational age, birth weight, pre- and periconceptual multiple micronutrient supplementation status, SES, and intrauterine environmental exposure, such as lead exposure and tobacco exposure. It has been long assumed that “men and women are growing old at different rates, with the former being much quicker.” But regarding inherited traits versus life experiences, which one plays a bigger role in hastening age? We examined methylation age at birth in substantially newborns and found no evidence of male aging faster than that of female. However, Hannum et al. [17] measured methylation age in adult cohort aged 19 to 101 years and succeed to identify significant contributions of gender to aging rate: the methylome of men appeared to age faster (~4%) than women. So, considering these facts, we concluded that the external factors may cause decisive changes in the way of aging.

Analysis of ethnicity demonstrated that ethnic group differences in methylation age were present as early as birth. Infants from mixed race/ethnicity origin had significantly higher methylation age and higher frequency of fast aging rate than that in African-origin black people. These findings complement previous findings of remarkable differences between ethnicity and race in global genomic DNA methylation [31]. Results of the relationship between maternal socioeconomic status and infants' methylation age demonstrate that SES was negatively and significantly associated with methylation age at birth (*r*_*s*_ = −0.48, *P*_*s*_ = 0.005). Findings from King et al. [32] suggest that epigenetics may relate the lower maternal socioeconomic status to adverse obstetrical outcomes (i.e., low birth weight) and even adult-onset chronic diseases and conditions (i.e., obesity, diabetes, cardiovascular diseases, and cancers). Therefore, further studies are needed to explore epigenome responses to environment of our human society, laying great emphasis on the importance of early-life experience.

Substantial evidence suggests that maternal smoking during pregnancy plays a critical role in many poor obstetric outcomes, including stillbirth, spontaneous abortion, and fetal growth restriction [33]. Furthermore, recently published study provided the first evidence of a significant positive association between tobacco exposure during pregnancy and shortened fetal telomere length in a dose-response pattern [4]. Thus, it is a plausible hypothesis that tobacco exposure in intrauterine life increases the risk of at least some of these adverse pregnancy outcomes through accelerated fetal biological aging. In line with this study hypothesis we found that risk of fast aging was about 3 times higher (OR = 3.17; 95% CI = 1.05–9.56) in mothers who smoked versus the risk in nonsmokers.

The exact mechanisms by which nicotine exposure drives acceleration in the methylation age of unborn babies are generally not well understood, although they may be plausibly attributed to cellular defense exhausting [34]. Thus, present study tried to compare the effect of nicotine exposure on estimated abundance measures of naïve CD4+/CD8+T cells, memory and effectors T cells, and proportions of CD4+/CD8+ T cells, NK cells, plasma blasts, and B cells between exposed and unexposed groups. We were pleasantly surprised to find that infants exposed to tobacco in utero had higher proportion of plasma blasts but lower naïve CD8+ T cell abundances compared with nonexposed group at birth. In unrelated replication sample, nicotine exposed group also displayed significant reductions in the proportions of naive CD4+ and naive CD8+ T cells but not all studies support this association; for example, results of Almanzar et al. [35] did not reveal differences between lymphocyte subpopulations of smoking mother and nonsmoking mother and their newborns, respectively. However, our study provides preliminary evidence for the potential relationship between maternal cigarette smoking, fast ageing of unborn babies, and substantial loss of naive T cells in cord blood.

However, we should also be aware of three major limitations of present study. First, data were measured using two different platforms: the Illumina Infinium 27K and the more recent Infinium 450K platform. Analysis shows that mean methylation age of infants at birth is significantly lower in the Illumina Infinium 27K platform (compared to those samples measured in the Infinium 450K platform) (*P* < 0.001). But the results performed in the combined datasets are consistently observed when restricting the analysis to each one dataset. Second, the number of samples per dataset and variable of interest varied greatly. Third, unvaried models of methylation age (fast aging) cannot exclude or control confounding caused by other covariates of interest. Fourth, although the “epigenetic clock” is a well-known epigenetic biomarker of aging described by Horvath (Genome Biology 2013), he also described that the median absolute difference between DNAm age and chronological age is around 2.9 years, in an optimistic scenario. However, it is scientific and feasible to use this calculator to determine whether the DNAm age of cord blood with special history of exposure is consistently higher (or lower) than that without it. Finally, we also need to caution the reader that, as the study design is primarily an observational study, we cannot establish causality but can only provide this as a speculative model.

As far as we know, this study provides the first evidence that methylation age at birth was independent of the infant's sex but significantly influenced by race. In utero exposure to maternal smoking and lower socioeconomic status are two important aging accelerators. With replication in newly collected sample, we observed strong evidence that smoking exposure during pregnancy is related to higher prevalence of fast aging of unborn babies and substantial loss of naive T cells in cord blood, a premature sign of immune aging. All these in turn suggest a plausible hypothesis that accelerated methylation aging may underlie some of the well-documented impacts of maternal adverse exposure during pregnancy on offspring.

However, a lot of practical questions remain: (i) exact molecular mechanisms underlying in utero programming of methylation age and the directionality of the relationship between in utero adverse exposure, methylation age, immunosenescence, and later health outcomes; (ii) whether this fast aging phenomenon in utero persists into childhood and adulthood; (iii) the window to modify the programmed fast aging, considering the possibility to reverse epigenetic modifications.

Thus, a multilevel approach is needed to explore (i) longitudinal human studies incorporating the evaluations of immune and proinflammatory measurements during prenatal period and (ii) molecular, cellular, and appropriate animal studies. Nonetheless, present study is designed to serve as a first step, but a most critical and important first step, in understanding that biological age, such as DNA methylation predicted age, is similar to some other traits influenced by both the genetic and environmental factors and related to the developmental origins of childhood and adulthood health and disease risk.

## 5. Conclusions

Collectively, we employed a novel epigenetic biomarker of aging (“epigenetic clock”) to quantify and assess the variability in biological age at birth and to better understand how the aging rates before birth are influenced by exposure in intrauterine period by employing. Results demonstrated that the methylation age at birth was independent of the infant's sex but were significantly influenced by race. Furthermore, common perinatal exposure (such as maternal smoking and lower socioeconomic status) may be important aging accelerators and substantial loss of naive T cells may play a role in smoking-related fast aging phenomenon.

## Supplementary Material

Figure S1: Prenatal tobacco exposure data and various exposed and non exposed group of cells abundances measurment data was analyzed but no significant differences were identified. Figure S2: Non smoking and smoking mother's newborns lymphocyte subpopulation of their cord blood samples data were analyzed and no significant difference were detected.

## Figures and Tables

**Table 1:** Summary details of the DNA methylation datasets from cord blood.

ID	Tissue sample	Methylation array	*n*	Availability	Average age acceleration (years)	Citation
1	Cord blood	Illumina 27K	168	GSE27317	−0.03 ± 0.29	Adkins et al. [7]
2	Cord blood	Illumina 27K	56	GSE34257	0.67 ± 2.85	Khulan et al. [8]
3	Cord blood	Illumina 27K	48	GSE36812	0.80 ± 1.08	Turan and Sapienza [9]
4	Cord blood	Illumina 27K	14	GSE35222	0.03 ± 0.23	Wang et al. [10]
5	Cord blood	Illumina 27K	216	GSE64940	0.18 ± 1.23	Mozhui et al. [11]
6	Cord blood	Illumina 450K	19	GSE30870	0.03 ± 0.13	Heyn et al. [12]
7	Cord blood	Illumina 450K	2	GSE37966	0.06 ± 0.002	Beyan et al. [13]
8	Cord blood	Illumina 450K	24	GSE54399	0.28 ± 0.35	Hughes et al. [14]
9	Cord blood	Illumina 450K	20	GSE64316	0.01 ± 0.17	Ivorra et al. [15]
10	Cord blood	Illumina 450K	46	GSE69636	1.94 ± 0.69	Sen et al. [16]

**Table 2:** Factors associated with positive age acceleration according to epigenetic age calculator among newborns.

Variables	Age acceleration		*β*	S.E.	Wald	*P* value	OR (95% CI)
	Positive	Negative					
Gender							
Male	237 (56.2%)	185 (43.8%)					1.00
Female	109 (57.1%)	82 (42.9%)	0.04	0.18	0.04	0.83	1.04 (0.74*–*1.47)
Race							
Africa-American	79 (38.7%)	125 (61.3%)					1.00
European-American	74 (46.0%)	87 (54.0%)	0.30	0.21	1.93	0.16	1.35 (0.89*–*2.05)
Mixed race group	12 (63.2%)	7 (36.8%)	1.00	0.50	4.03	**0.04**	**2.71 (1.02–7.18)**
Supplementation status							
Placebo	24 (68.6%)	11 (31.4%)					1.00
Micronutrient	11 (52.4%)	10 (47.6%)	−0.68	0.57	1.45	0.23	0.50 (0.17*–*1.54)
Tobacco exposure							
No	24 (66.7%)	12 (33.3%)					1.00
Yes	38 (86.4%)	6 (13.6%)	1.15	0.56	4.18	**0.036**	**3.17 (1.05–9.56)**
