# Supplementary figures and images for: Infant's DNA Methylation Age at Birth and Epigenetic Aging Accelerators
Source: Biomed Res Int. 2016 Dec 12;2016:4515928. doi: 10.1155/2016/4515928 (PMC5183755; doi:10.1155/2016/4515928)

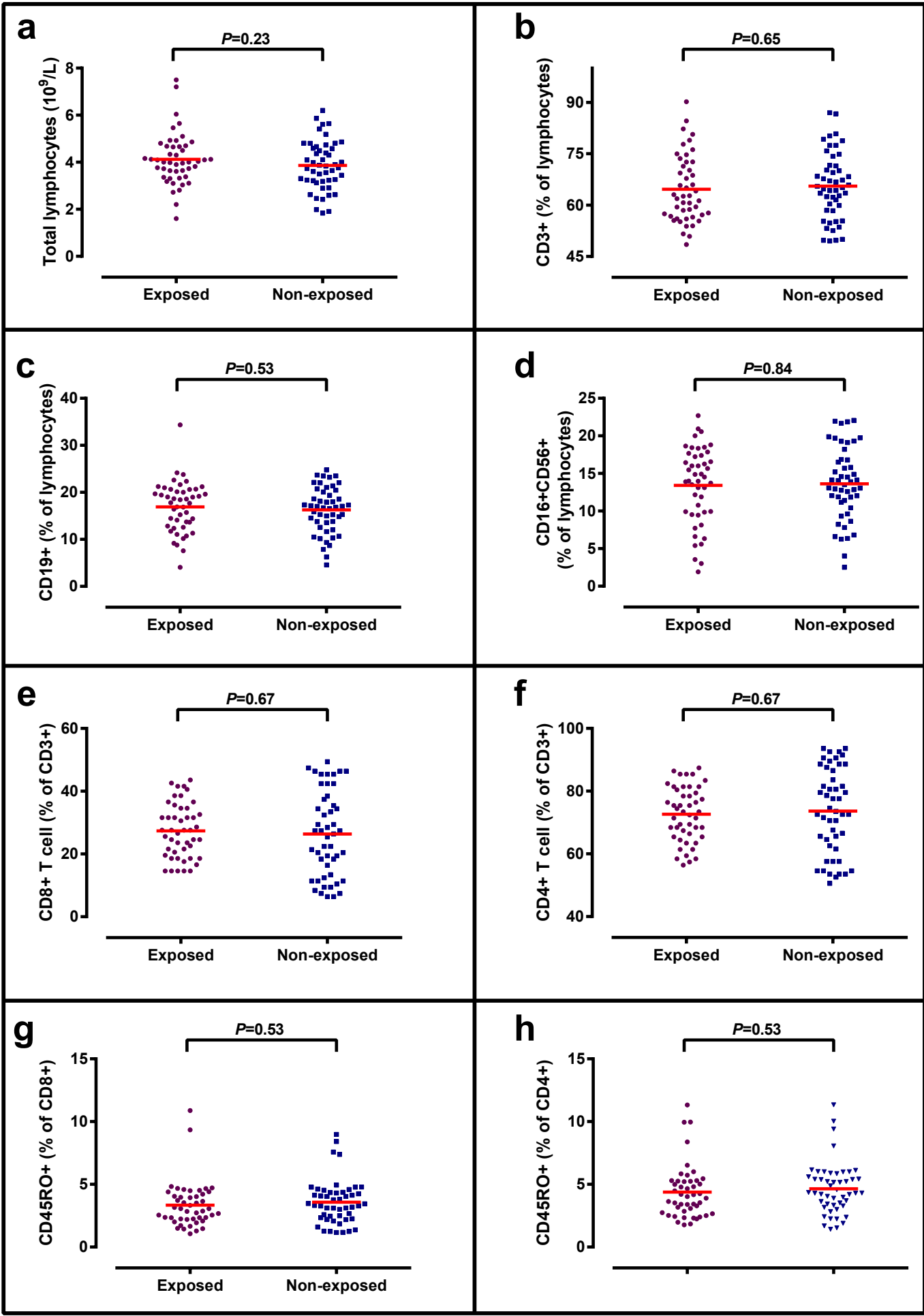

**Figure S2.** Lymphocyte subpopulations in newborns of smoking and non-smoking mothers.

Supplement: Supplementary file 1 — Figure S1: Prenatal tobacco exposure data and various exposed and non exposed group of cells abundances measurment data was analyzed but no significant differences were identified. Figure S2: Non smoking and smoking mother's newborns lymphocyte subpopulation of their cord blood samples data were analyzed and no significant difference were detected. [file 4515928.f1.zip › 4515928/Figure S2_correct.pdf]
